# Unraveling Anastomosing Hemangioma: A Case Report

**DOI:** 10.7759/cureus.55351

**Published:** 2024-03-01

**Authors:** Brayan Muñoz-Caicedo, Vanessa García-Gómez, Carolina Gutiérrez, Brian Noreña-Rengifo, Jack Muñoz-Caicedo

**Affiliations:** 1 Radiology, Universidad de Antioquia, Medellín, COL; 2 Radiology, Hospital Pablo Tobón Uribe, Medellín, COL; 3 Radiology, CediMed, Medellín, COL

**Keywords:** retroperitoneal tumour, anastomosing hemangioma, retroperitoneal, ct, mri

## Abstract

Anastomosing hemangioma is a rare and benign subtype of capillary hemangioma, a soft tissue tumor. It tends to be asymptomatic, causes abdominal pain and hematuria, and is more common in the genitourinary tract. It can be confused with paragangliomas or ectopic pheochromocytomas. Pathology shares characteristics with angiosarcoma, particularly in well-differentiated areas. Diagnosis without a surgical specimen is difficult and is based on clinical characteristics, laboratories, and imaging behavior similar to hemangiomas in other locations. When in doubt, a diagnosis can be supported by a percutaneous biopsy. The prognosis is good, without relapses or metastases. Early identification with follow-up can avoid surgical interventions.

## Introduction

Hemangiomas are benign tumors belonging to a heterogeneous group of soft tissue neoplasms and are known for having an abnormal proliferation of blood vessels. They can be cavernous or capillary. The histological subtype of anastomosing hemangioma (AH), classified as capillary hemangioma, was first described by Montgomery et al. [1] in 2009 in six patients with genitourinary tract lesions. However, it is still considered a rare entity, with 60 cases published by 2021. Nevertheless, AH was recognized in the 2020 WHO classification of soft tissue tumors [2-6].

Patients with AH tend to be asymptomatic and have an association with renal failure. Despite its benign nature, many AHs end up requiring surgical resection, a step that could be avoided with careful consideration of this diagnosis [4]. Differential diagnoses are considered related to the location of the AH and include angiosarcoma, clear-cell renal cell carcinoma, ectopic paraganglioma, or pheochromocytoma, among others [1,4,5].

In imaging, AH is characterized by circumscribed benign-appearing lesions with progressive enhancement and paravertebral retroperitoneal location [7]. In pathology, it appears as densely branched and anastomosed sinusoids covered by endothelial cells. On immunohistochemistry, it is positive for the markers CD31 and CD34 [8]. When these features are lost, the differential diagnoses must be contemplated. This research presents a case of retroperitoneal AH with pathology proven in surgical resection. It contributes to the literature through the images of CT, but especially MRI, which are not frequently included.

## Case presentation

**Figure 1:**
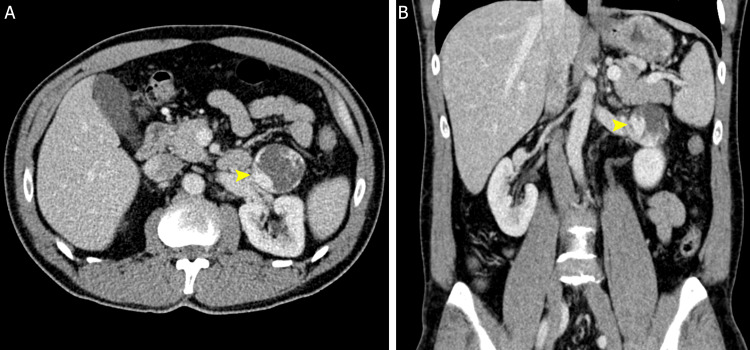
Abdominal CECT portal venous phase, axial plane (A), and coronal reconstruction (B) Located in the left perirenal space and anterior to the renal vessels, a round circumscribed mass (yellow arrows) measuring 4.5 cm x 4 cm (transverse x anteroposterior) with discontinuous peripheral nodular enhancement and a center of lower density for the acquisition phase. No phleboliths were observed in the mass, and there were no signs of thrombosis, vascular invasion, adenomegaly, or metastatic lesions. CECT: Contrast-enhanced computed tomography

A 54-year-old male patient with a history of hypertension, benign prostatic hyperplasia, and sticky platelet syndrome consulted the emergency department for ill-defined chronic abdominal pain. After the initial clinical evaluation, it was not possible to find the reason behind the pain, so an abdominal CT with intravenous contrast was performed (Figure 1).

**Figure 2:**
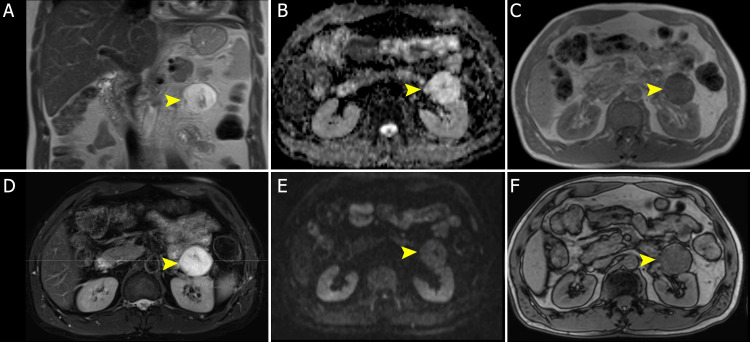
Abdominal MRI part 1 Coronal T2-weighted sequence (A) and axial fat-suppressed T2-weighted sequence (D) show a very high signal-intensity mass, similar to the one from cerebrospinal fluid. The mass has no diffusion restriction in the ADC map (B) and the b800 DWI sequence (E). The mass has no signal drop in the dual-echo in- (C) and opposed-phase T1-weighted imaging (F). ADC: Apparent diffusion coefficient, DWI: Diffusion-weighted imaging

**Figure 3:**
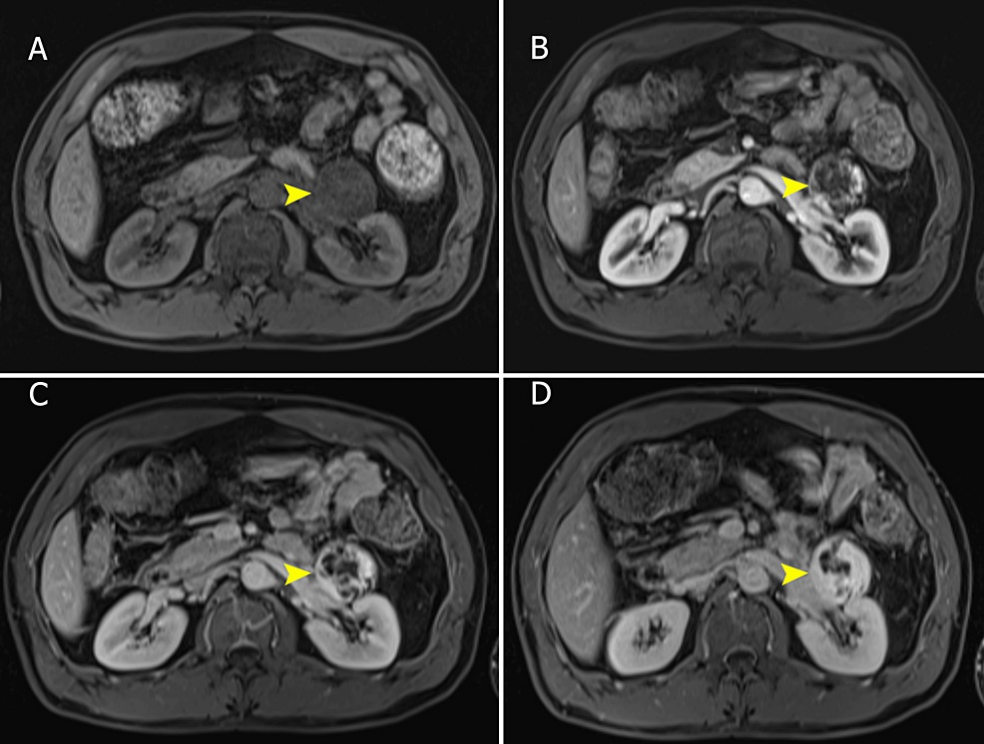
Abdominal MRI part 2, dynamic sequences Simple-phase fat-suppressed T1-weighted MRI sequence (A) and dynamic sequences after intravenous contrast medium administration (B, C, and D) show a low-signal-intensity mass in the simple phase and an avid, nodular, peripheral, and discontinuous enhancement in the corticomedullary phase, which is progressive and centripetal over time. The size of the lesion remained stable compared to the previous CT.

Among the main differential diagnoses considered for the tomographic findings were exophytic renal angiomyolipoma, pheochromocytoma (hypertensive patient), and AH. Since the blood biochemical and clinical evaluation did not suggest a significant alteration of the function of the adrenal gland, an echoendoscopic biopsy was performed due to its proximity to the stomach, but it was not possible to obtain a significant sample for the diagnosis, so an abdominal contrast MRI was carried out to provide a better characterization of the mass and as a presurgical assessment for possible resection (Figures 2-3).

**Figure 4:**
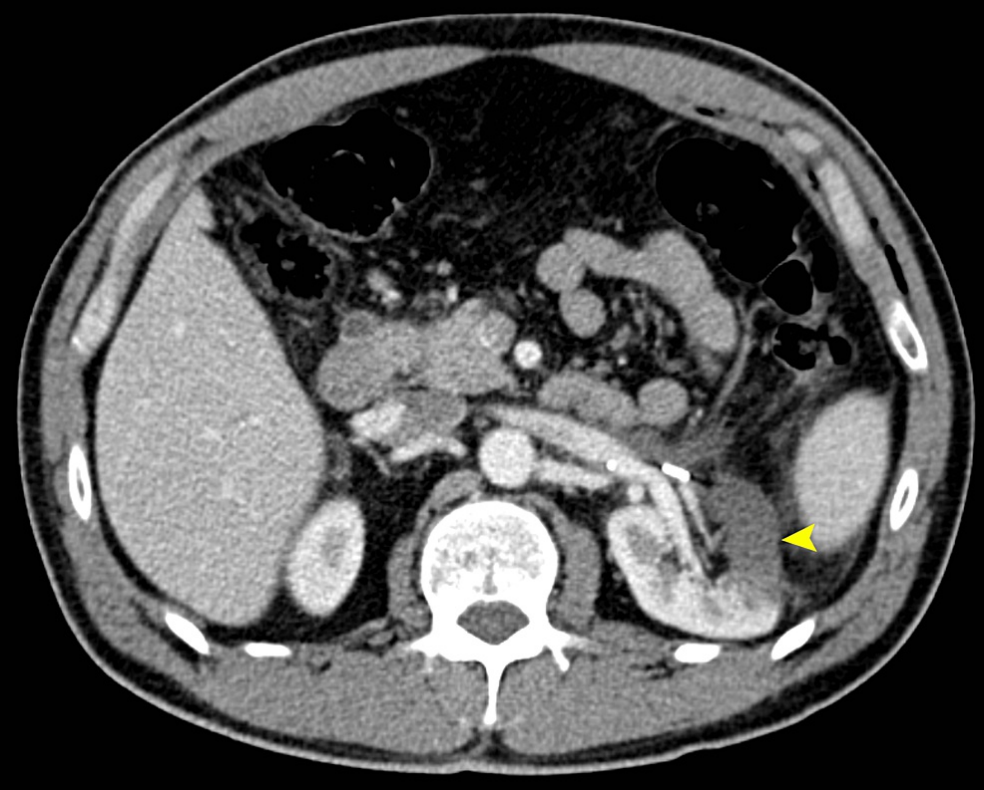
Abdominal CECT in corticomedullary phase, axial section over the renal hilum Observed is a wedge-shaped hypodensity over the anterior region of the left kidney due to segmental infarction. CECT: Contrast Enhanced Computed Tomography

The patient was taken for surgical resection, where it was found that the mass was adherent to the renal hilum vascular structures, mainly the renal vein. After surgical resection, a histologic analysis with immunohistochemistry confirmed the diagnosis of AH with CD31(+) and CD34(+) markers. The patient presented with a postoperative segmental left renal infarction (Figure 4). There were no other complications or signs of recurrence during institutional follow-up.

## Discussion

According to the WHO, AH is a benign vascular tumor recognized as a distinct entity that can be classified according to its renal or extrarenal location. Almost half of the cases are in the paravertebral retroperitoneal region and the renal space (including the perirenal). Isolated renal AH represents 22% of the cases. Anastomosing hemangioma can also be found in the adrenal glands, spermatic cord, testes, ovaries, liver, colon, small intestine, mesentery, bone, breast, nasal cavity, left atrium, and others [3,4,7].

The age range of patients is highly variable, affecting any age group from eight to 85 years old in the reported cases. However, the average age of presentation for renal AH is 49 years old, with a male predominance of 2.3 times; for extrarenal AH, the average age is 65 years old, also being 1.3 times more likely in men [4]. Clinically, a factor associated with renal AH is renal dysfunction (in two out of three patients), and it is usually discovered as an incidental imaging finding [4,8]. Other patients may report low back pain, hematuria, subcapsular hematoma, or mass effect symptoms according to location and size [7,9]. 

In imaging, AH presents as a nodule (<3 cm) or confined, rounded mass (≥3 cm). It is generally bigger than 2 cm at the time of the radiologic study. The lesion tends to be unique, although bilateral and multifocal cases have been described. In ultrasound, AH is hypoechoic in B-mode with a hypervascular signal for color Doppler. In CT, they are dense (more than 50% of the Hounsfield units (HU) of the psoas muscle or between 27 and 35 HU), hypervascular with avid enhancement, which can be heterogeneous or peripheral, nodular, and discontinuous. In MRI, the signal tends to be hypointense in the T1 weighted image (T1WI), markedly hyperintense in the T2WI, slightly hyperintense in diffusion-weighted imaging (DWI), and fatty or cystic changes may be identified in specialized sequences. Imagenologic findings are not specific, which complicates presurgical diagnosis. If there are lesions in other organs, it becomes more difficult to suspect this entity. Even with stable follow-up, it cannot be diagnosed reliably [3-5,7].

Differential diagnoses for renal AH include renal cell carcinoma clear-cell subtype. For extrarenal AH, ectopic paraganglioma or pheochromocytoma is considered, especially for hypervascular retroperitoneal or adrenal gland lesions. Angiosarcoma is another diagnosis to consider. However, the AH does not display an infiltrative aspect, meaningful atypia, mitotic activity with high proliferation rates, multilayer formations, or necrosis. The well-differentiated areas of the angiosarcoma with a similar appearance to AH require special mention [1,4]. Kaposi sarcoma is a pathologic consideration in lesions with increased plasmatic and non-pleomorphic cells and positive lesions to herpesvirus type 8 [4]. Other possibilities are liposarcoma, solitary fibrous tumor, Schwannoma, or accessory spleen [5,8].

In pathology, the macroscopic aspect is a confined, spongy lesion with a soft texture, brown color, or hemorrhagic zones. Microscopically, the most important characteristics are vascular branched anastomosed structures covered by endothelial cells, some in the hobnail, hyaline globules, and extramedullary hematopoiesis of the principal lines. There are zones with capillary thrombosis and renal tubular entrapment [4,5,8,9]. The differentiation of angiosarcoma is also difficult. For this reason, it leans on immunohistochemistry, where AH vascular lining cells have positive markers for CD31 and CD34 and are negative for CD8, D2/40, GLUT-1, AE1/AE3, EMA, SMA, HMB-45, Melan-A, and HHV-8; Ki67 is low [8,9].

In most cases, nephrectomy has been reported as a surgical treatment [1]. But in an adequate clinical scenario, a Tru-Cut percutaneous biopsy under suspicion of AH has been described by O'Neil et al. [7] in seven out of 32 patients included in the study without any report of associated hemorrhagic complications, an approach proposed to avoid surgery [5,7]. Thus, observation or embolization are treatment options [9,10].

Since the behavior of AH is benign, their prognosis is excellent, with no reports of recurrence or metastases in those where resection was performed [4]. However, since the cases in the literature are scarce, it is necessary to continue reporting and researching to refine the findings to improve patients' diagnosis and long-term follow-up, impacting potential evidence-based management without compromising patient safety.

A limitation in the diagnostic process for this case report was that it showcases only the initial portal-venous phase of the abdominal CT. There were no other CT phases available, making it impossible to probe the dynamic and progressive enhancement, which was a constraint. However, the imaging of MRI is an advantage in this case because it is not frequently included in reports and adds value to the literature. And, as a case report, another limitation is the representation of a single case with an appearance and enhancement behavior similar to hemangiomas in other organs, the liver, for example. Thus, these results cannot be translated for atypical cases with variable enhancement.

It is necessary to familiarize ourselves with other patterns of AH and increase detection with broader reports. But, for rare entities like this one, this format of publication might be the best option to share experiences from different hospitals around the globe and, in time, provide enough material to make precise statements.

## Conclusions

Anastomosing hemangiomas are benign, distinctive, and rare vascular tumors that occur in any age group and with variable locations. It is usually an incidental finding in diagnostic imaging with radiologic features that may be reminiscent of hemangiomas in other organs or, to be less specific, cases where a prospective diagnosis is more complicated. When clinical suspicion, laboratory tests, and imaging are consistent with AH diagnosis, percutaneous biopsy can be used to pursue non-surgical management with follow-up. Angiosarcoma is the principal differential diagnosis based on their similarity, even from a pathological viewpoint. This entity has an excellent prognosis without relapse or metastasis.
